# Severe Hypernatremia Due to Postoperative Diabetic Ketoacidosis and Transient Central Diabetes Insipidus: A Case Report

**DOI:** 10.7759/cureus.104400

**Published:** 2026-02-27

**Authors:** Bilal A Mohammed, Rana Fatima, Anila T, Challa Parameshwar, Uday Kumar K

**Affiliations:** 1 Emergency Medicine, Mythri Crystal Hospital, Hyderabad, IND; 2 Nephrology, Mythri Crystal Hospital, Hyderabad, IND; 3 Gastrointestinal Surgery, Mythri Crystal Hospital, Hyderabad, IND; 4 General Surgery, Mythri Crystal Hospital, Hyderabad, IND; 5 Anaesthesiology, Osmania Medical College, Hyderabad, IND

**Keywords:** central diabetes insipidus, diabetic ketoacidosis, hypernatremia, postoperative state, slow low efficiency dialysis

## Abstract

We report a case of acute severe hypernatremia secondary to diabetic ketoacidosis (DKA) and transient central diabetes insipidus (CDI) in a postoperative patient. Diagnosis of DKA may be delayed in patients on sodium-glucose cotransporter 2 (SGLT2) inhibitors, particularly in the postoperative state. Hypernatremia was refractory to medical management and corrected with slow low-efficiency dialysis (SLED) and desmopressin supplementation. This case highlights the importance of suspecting diabetes insipidus in refractory hypernatremia and demonstrates the role of SLED for controlled sodium correction in unstable patients.

## Introduction

Euglycemic diabetic ketoacidosis (DKA) associated with sodium-glucose cotransporter 2 (SGLT2) inhibitors is prone to diagnostic delay, especially in the postoperative period [1,2]. Severe dyselectrolytemias in DKA may arise from both the metabolic derangements of ketoacidosis and its management [3]. Hypernatremia is an uncommon but serious complication, typically resulting from osmotic diuresis and inappropriate sodium bicarbonate therapy and decreased water intake in postoperative patients [4,5]. We present a rare case of postoperative DKA with refractory hypernatremia, attributed to a diagnostic overlap with transient diabetes insipidus due to vasopressin deficiency.

## Case presentation

A 67-year-old man with diabetes had undergone right hemicolectomy with ileocecal anastomosis for carcinoma cecum. Preoperative glycemic control was poor (HbA1c, 8.47%). His anti-hyperglycemic medications were Insulin glargine, glimepiride, dapagliflozin, metformin, and sitagliptin. Surgery was uneventful.

On postoperative day (POD) 3, he developed lethargy and nausea, and on POD 5, he became drowsy, afebrile, tachypneic (respiratory rate (RR) 24 breaths per minute, oxygen saturation (SpO2) 98 % at room air, and low blood pressure (BP) 90/60 mmHg). His systemic examination was normal. Random blood sugar was 203 mg/dl. Arterial blood gas (ABG) analysis revealed severe metabolic acidosis (pH 6.99, bicarbonate (HCO3) 1.4) with an anion gap of 28.2 mmol/L. Urine ketones were strongly positive. Serum beta-hydroxybutyrate levels were elevated (5.840 mmol/L). Electrocardiography, serum cardiac enzymes, and two-dimensional echocardiography did not show any acute changes suggestive of acute coronary syndrome (Table 1).

**Table 1:** Laboratory parameters during hospital stay POD: postoperative day; WBC: white blood cell; ABG: arterial blood gas; PCO2: partial pressure of carbon dioxide; HCO3: bicarbonate; FBS: fasting blood sugar

Investigations	Day of surgery	POD 5	POD 8	POD 9	Day of discharge
Hemoglobin (gm/dl)	9.2	10.3	-10.8	11.2-	11.0
WBC (/mm^3^)	8300	15,400	18,000	14,000	9400
Urine ketones	Negative	Strongly positive	Strongly positive	Negative	Negative
ABG pH	7.34	6.99	7.19	7.49	7.35
PCO2	38	5.8	16.7	15.9	39
HCO3	22	1.4	6.2	11.9	21
FBS (mg/dl)	76	230	207	128	155
Serum creatinine (mg/dl)	0.62	1.1	1.2	1.4	1.4
Serum sodium (mmol/L)	136	143	172	165/161	146
Serum potassium (mmol/L)	5.2	4.8	3.4	3.2/2.9	3.6
Serum chloride (mmol/L)	102	110	122	128/121	115

DKA was treated with Intravenous (IV) infusion of insulin, normal saline, and sodium bicarbonate. His antibiotics were stepped up. Serum sodium (Na) was elevated (154 mmol/L), and he had excessive thirst with polyuria. Free water supplementation was given (IV dextrose 5% in water (D5W) 100 ml/hour and oral route-continuous Ryle's tube water infusion 50-100 ml/hour). Na increased to 170 mmol/L despite ongoing correction. Slow low-efficiency dialysis (SLED) with high-sodium dialysate bath was prescribed for six hours with dialysate sodium profiling, and NA was checked every two hours of the procedure.

Na levels reduced to 162 mmol/l within two hours of SLED. The procedure was stopped to prevent complications related to rapid correction, and free water correction was continued. Urine osmolality (Uosm) was inappropriately low (390 mOsm/Kg) for serum hyperosmolarity of 359 mOsm/Kg and indicates a diabetes insipidus state. Hence, a therapeutic trial with desmopressin was initiated. An MRI of the brain was not done due to clinical instability. The water deprivation test was contraindicated. Serum cortisol (14.4 IU/L) and serum thyroid-stimulating hormone (TSH) (1.6IU/L) were normal. Desmopressin nasal spray 10 mcg was given every 12 hours for four days. There was a reduction in urine output (<2 litres/day) and a gradual decrease in Na levels.

The patient was discharged on the 12th postoperative day.

## Discussion

The relative fasting period (preoperatively due to gastrointestinal malignancy and postoperatively due to diet restriction), the surgical stress, and sub-optimal insulin supplementation and SGLT2 inhibitors usage may have all precipitated DKA [1-3]. There should be a high suspicion of (euglycemic) DKA in ‘sick’ postoperative patients on SGLT2 inhibitors. Hypernatremia in DKA occurs due to polyuria and sodium bicarb supplementation. Persistent hypernatremia in spite of free water replacement, ongoing polyuria, and inappropriately low urine osmolality raised the suspicion of diabetes insipidus [6]. DI was transient and resolved within five days, partial as there was no other pituitary hormonal deficiency (TSH and cortisol were normal), and central or vasopressin-deficient as seen by the low urine osmolality in the background of high serum osmolality and response to desmopressin.

This ‘transient partial central’ DI in our patient was probably due to temporary ‘stress-related dysregulation’ in antidiuretic hormone (ADH) secretion precipitated by surgery per se and later by hypovolemia, hypotension, and severe acidosis. Similar rare cases had been reported in which transient central diabetes insipidus was seen in non-pituitary postoperative states and also non-surgical critical cases [7-11].

A short course of desmopressin helped in salvaging the crisis, indicating a delayed and suboptimal response by the posterior pituitary to the hyperosmolarity stimulus. Whether a short therapeutic trial of desmopressin may salvage acute severe hypernatremia in selected cases is uncertain and warrants further study [12]. This case can be conceptualized as a state of diabetes neuter, representing the coexistence of diabetes mellitus and diabetes insipidus. This case also demonstrates the lifesaving benefit of timely SLED for safe Na correction in a hemodynamically unstable patient with severe hypernatremia.

## Conclusions

This case illustrates a rare cause of refractory hypernatremia in postoperative diabetic ketoacidosis due to transient central diabetes insipidus. Early recognition of inappropriate polyuria and low urine osmolality is essential. Slow low-efficiency dialysis with Na profiling allowed safe correction of severe hypernatremia in a hemodynamically unstable patient, while a short course of desmopressin effectively reversed the diabetes insipidus. Awareness of this entity can prevent catastrophic neurologic complications in similar ICU settings.
